# An Approach to Task Representation Based on Object Features and Affordances

**DOI:** 10.3390/s22166156

**Published:** 2022-08-17

**Authors:** Paul Gajewski, Bipin Indurkhya

**Affiliations:** 1 Institute of Computer Science, AGH University of Science and Technology, 30-059 Krakow, Poland; 2 Institute of Philosophy, Jagiellonian University, 31-007 Krakow, Poland

**Keywords:** computer vision, explainability, task understanding, scene understanding, robot perception

## Abstract

Multi-purpose service robots must execute their tasks reliably in different situations, as well as learn from humans and explain their plans to them. We address these issues by introducing a knowledge representation scheme to facilitate skill generalization and explainability. This scheme allows representing knowledge of the robot’s understanding of a scene and performed task. We also present techniques for extracting this knowledge from raw data. Such knowledge representation and extraction methods have not been explored adequately in previous research. Our approach does not require any prior knowledge or 3D models of the objects involved. Moreover, the representation scheme is easy to understand for humans. The system is modular so that new recognition or reasoning routines can be added without changing the basic architecture. We developed a computer vision system and a task reasoning module that works with our knowledge representation. The efficacy of our approach is demonstrated with two different tasks: hanging items on pegs and stacking one item on another. A formalization of our knowledge representation scheme is presented, showing how the system is capable of learning from a few demonstrations.

## 1. Introduction

One issue with deploying multi-purpose service robots in human-occupied spaces, such as homes, is that these environments are constantly changing. Therefore, to function well, robots must be robust to changes in the environment or situation. Another issue is that the robot’s workspace is shared with humans, who might tell the robot what to do, and who need to understand the intentions of the robot to feel safe and comfortable around it [1]. These issues (i.e., adaptation and communication) can be addressed by designing appropriate knowledge representation. We present such a knowledge representation formalism here to facilitate both skill generalization and explainability. To deploy this knowledge representation in practice, we developed a set of techniques to extract knowledge automatically from raw input data, which are also presented here (the source code is available online at: https://github.com/lubiluk/halepensis (accessed on 12 August 2022)).

The foundation of our knowledge representation is a graph-based scheme for scene and task understanding, which can be conveniently viewed and interpreted by humans. The process of building such a graph can be divided into two stages. The first stage, which does not consider the goal of the task, finds objects, object features, and relations between them. As a result, a graph of spatial–visual concepts is built. In the second stage, which incorporates the goal of the task, an analysis of the scene graph is performed. It infers what changes are made during the demonstration and which of these changes are essential for the task. At the end of this stage, we obtain knowledge about the task at hand in a compact form: as a graph and operations on the graph.

Details, such as feature grounding or trajectory calculation, are left to be inferred during the task execution. We show that by capturing only essential details, our compact representation is particularly suited for generalization and improvization. Just as humans can improvise and repurpose an object for a different use, such as using a mug to scoop some soup out of a container [2], our system can also consider the task requirements and affordances of the available objects to adopt an object for a novel use to fulfill a given task. Moreover, in our approach, task completion criteria are encoded right in the knowledge representation, so we can check if our actions have succeeded or not, and keep trying until the desired goal is reached.

Many contemporary semantic task representations encode information in hierarchical ontologies [3,4]. These facilitate generalizations where we can infer that an object can be used in a certain way based on the category to which it belongs. For instance, a cup belongs to the category vessel, so it can be used for drinking, mixing, or pouring [4]. However, such ontologies suffer from one significant drawback: objects and their categories must be decided beforehand. The robot must accurately recognize an object from the input data, and place it in an appropriate category, which constrains how it can be used. So, though a robot can infer that a cup can be used for carrying tea (for instance), it cannot infer that the cup can be used as a projectile to throw at a burglar.

In contrast to most other works that design knowledge representations around objects [3,4,5,6], our approach decomposes objects into constituent features that facilitate certain actions, a method that is motivated by biological models of how the human brain learns object affordances [7,8]. For example, a “virtual finger” hypothesis has been proposed [9] for ad hoc motion planning to satisfy the task constraints obtained by analyzing the current situation.

Another aspect of task representation is describing a motion sequence that achieves the desired goal. Previous research studies have used pre-programmed routines [2,3], trained control modules [10], or other forms of motion notations [11] to describe motion trajectories, and assign meanings to particular actions, such as “picking up”. In our approach, we assume that motion categories or even trajectories are not crucial for task descriptions. There might be a number of possible trajectories that can achieve the desired results, and their particular manifestations largely depend on the anatomy of the robot and the available spatial constraints. Therefore, we opt for motion plans that are calculated ad hoc: on the spot where the motion is needed, and at the time when the motion is needed. Appropriate motion planners can then be put in place to be used when accurate data are available [12,13].

The efficacy of our approach—focusing on object features and generating motion trajectories on the fly—was demonstrated in a robotic system prototype in our previous research [2]. In this earlier approach, useful object features needed to be hand-picked. In the research presented here, we extended this approach to infer object features automatically.

We explain here the principle of our knowledge representation formalism, and present algorithms based on it in a simplified scenario where the input to our reasoning algorithm consists of two point clouds: one presenting the scene before the task execution, and the other after taking a sequence of actions. The system analyzes these two point clouds and generates a description of the actions that comprise the task in the formalism of our representation.

The novelty of our approach lies in: (a) representing objects as a set of constituent features or affordances, (b) system transparency for greater explainability, and (c) working with objects without having their full 3D models upfront.

Due to the novelty of our task understanding representation, and the fact that we propose a new way of generalizing knowledge, our method is not directly comparable, in terms of performance, to any of the existing state-of-the-art approaches. Therefore, to demonstrate the efficacy of our approach, we present qualitative experimental results on two different tasks.

The rest of the article is organized as follows: First, we review the related research, then we introduce our knowledge representation scheme and the design of a system built around it. We present the formalization of our knowledge representation and algorithms and conduct the computational complexity analysis. Next, we discuss experimental evaluation results from two tasks: the task of hanging items on pegs, and the task of stacking items. Afterward, we perform ablation studies of our reasoning system. Finally, we present our conclusions and discuss future research directions.

## 2. Related Work

The problem of a scene and task understanding is directly connected to the one of robotic skill adaptation. Robotic skill representation and adaptation is a major topic in robotic research with a number of different approaches present in the literature, though the problem is far from being solved. Some contemporary approaches are based on deep learning [10,14,15] or probabilistic [16,17] methods to encode a skill completely. Even though they often achieve good performance and generalization capabilities, they are not able to explain their decisions to human users, because their knowledge is encoded in the opaque form of neural network weights or probabilities. In this work, we explore a knowledge representation system based on symbols that is understandable and can be interpreted by a human. Works that are most closely related to our research are those that focus on symbolic and semantic knowledge representations, such as semantic graphs [17] or ontologies [3,4,18,19,20,21,22].

Knowledge representation is recognized as a key ingredient in a successful robotic system, especially when the robot is intended to work among humans or in human environments [23,24]. Knowledge representation also lies at the heart of the most proposed solutions to scene understanding, which are comparable to our approach. Good knowledge representation should undoubtedly be robust, meaning that it should allow the robot to generalize its skills. Explainability of the robot’s knowledge and its decisions is also desired if the robot is to be deployed in human-occupied spaces.

Many existing approaches to these problems rely on ontologies as the basis for knowledge representation. An ontology is a tree-like structure containing information about objects and places. By traversing an ontology we can find answers to commonsense questions, such as: what kind of utility class does the object at hand belong to; or what can be done with this object, i.e., what are its typical affordances. A notable ontology often used in related research is KnowRob [4], which is based on an earlier Open-EASE project [25]. It lets a user, typically a robot, query various types of information about surrounding objects. Alternate solutions, such as knowledge embeddings [26], serve the same purpose of finding information about objects. However, all information in such ontologies is pre-compiled earlier (for instance) by scraping websites or available databases. Moreover, known objects have full 3D models available. In a robotic setup, the knowledge base is often accompanied by a separate system for describing perceived situations and task plans, and for governing plan execution, as knowledge coming from ontologies or other sources must be combined with the raw data available to the robot in order to be used for scene understanding or manipulation [27,28,29,30].

Most of the related works treat objects as whole entities and assign roles to them. However, some works consider constituent object features as the bases for reasoning. For example, [6] decomposes objects into constituent features, but focuses only on features related to one particular action, namely grasping. In our approach, we discover multiple features so that features relevant to different actions can be selected. In [31], object affordances are considered as functions of object parts and their spatial relations. However, they focus only on in-hand tool classification, whereas we implement a similar idea on a much broader scale.

To illustrate how our system advances the state-of-the-art, we compare our system with other closely related ones in Table 1. None of the existing systems perform exactly the same task as ours, but we can compare symbolic knowledge representations as well as learning abilities of related systems. We can see from the table that our system incorporates some of the most desirable properties, such as working without 3D models of objects, learning efficiently, and generalizing to different tasks. Moreover, our system provides human-understandable representation of its scene and task understanding.

In this research, we draw inspiration from cognitive science ideas, such as the idea of projection as a tool for skill adaptation [32]. Features in our system are not rigidly tied to particular objects but are discovered on the fly, opening up a possibility for reinterpreting scenes depending on the task at hand.

## 3. System Overview

### 3.1. A Graph-Based Knowledge Representation

Our knowledge representation is based on graphs. A set of graphs represents all the knowledge discovered by the system about the situation at hand. There is one graph for each step of the task, of which there are currently only two: before hanging the item and after hanging the item. The result of the reasoning system, which captures the knowledge about task requirements, is also presented as a graph.

Each node in the graph represents a physical part or property of the scene; therefore, we call these nodes “entities”. There are two types of nodes: one for objects and one for everything else that belongs to objects. Therefore, an object is always a top-level node in the graph. Internally, entities carry a set of data useful for different parts of the recognition and reasoning system: type, ID, 3D position and orientation, bounding box corners, and a point cloud. The list of all possible relation types is predefined, but can be changed, and consists of all types of entities for which there are recognizers.

Edges represent relations between entities. The basic and most prevalent relation used is that of containment and is expressed as a “has” relation. For visual clarity, we omit labels for such relations. Edges carry only one type of information, namely the relation type. Similar to the entity types, the list of all possible relation types is predefined but can be changed. Each new revision of the system may add new relation types; with each relation type accompanied by an appropriate relation recognizer.

Our system is designed to be used by a robot to learn from a human demonstration of how to perform a certain task. We implemented a prototype of our system for the use case of hanging items on pegs. After a training round of demonstrations, the robot is able to hang arbitrary objects in different types of hangers, as long as the object has some affordances for hanging, e.g., some kind of aperture or a curved hook. The robot also positions the object correctly, and with stability, so that it stays hung without falling or swinging. This requires that the item be hung by an aperture above its center of mass. All of this information is incorporated into the knowledge generated by our system.

### 3.2. Task-Understanding Algorithm

The algorithm for task understanding proceeds in the following six steps:

Step 1: Pre-processing the raw input point clouds.
Step 2: Detecting objects in the scene.
Step 3: Detecting changes between scenes.
Step 4: Detecting features of objects that participated in the change.
Step 5: Describing spatial relations between features.
Step 6: Forming hypotheses about key task requirements.

**Figure 1:**
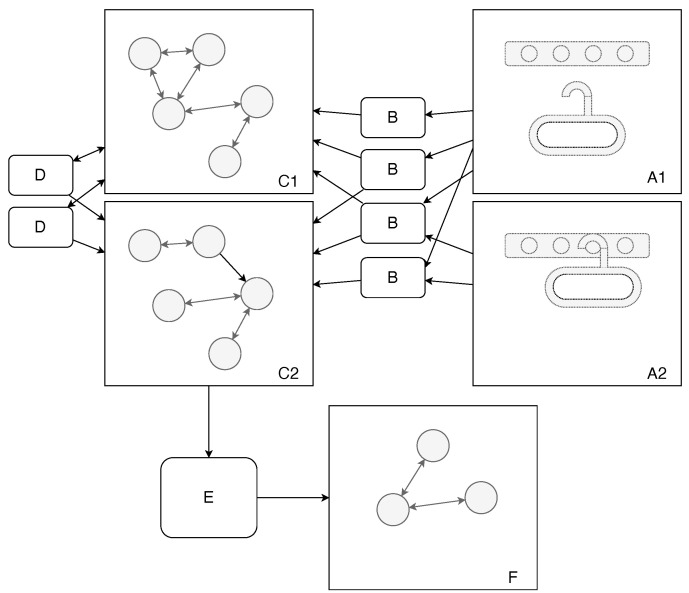
System overview. **A1**—point cloud before task execution; **A2**—point cloud after task execution; **B**—feature detection routines; **C1**—scene understanding graph before task execution; **C2**—scene understanding graph after task execution; **D**—relation detection routines; **E**—reasoning module; **F**—task description.

Results from Steps 1–4 are stored in a knowledge graph. Each consecutive step, except the first, uses information available in the graph. Figure 1 provides a graphical overview of the algorithm.

The steps of the algorithm result in a task-understanding graph. However, if the procedure is applied at least twice to slightly different situations, we can merge their resulting task-understanding graphs into a generic skill-knowledge graph. We now describe below each step of the algorithm.

### 3.3. Pre-Processing the Input Point Clouds

Before the scene understanding pipeline begins its work, the input point clouds are pre-processed. Raw point clouds come from an Intel RealSense L515 LiDAR camera. This camera typically produces a small number of artifact points between objects that are at different distances from the camera. Those points are removed using statistical outlier removal [33]. As the hanging task takes place near a wall, a significant number of points in raw point clouds belong to the wall. These points are removed using RANSAC plane segmentation [34]. In the case of the stacking task, the same method is used to remove the table plane.

### 3.4. Detecting Objects

The next step of the algorithm is to find distinct objects. This is done using the Euclidean cluster extraction [35] for the before-scene. Later, the same objects are detected in the after-scene using the iterative closest point aligning algorithm. It is crucial to keep track of which objects from the before-scene correspond to which objects from the after-scene for the later steps to work properly.

### 3.5. Detecting Changes

To reduce the number of expensive recognition and detection operations, only a subset of the objects in the scene is considered for the analysis. The algorithm focuses on objects that have moved or are in contact with a moving object. This is done by comparing object positions before and after the task execution. Holding the camera firmly in place while performing the task demonstration facilitates this process.

### 3.6. Detecting Object Features

**Figure 2:**
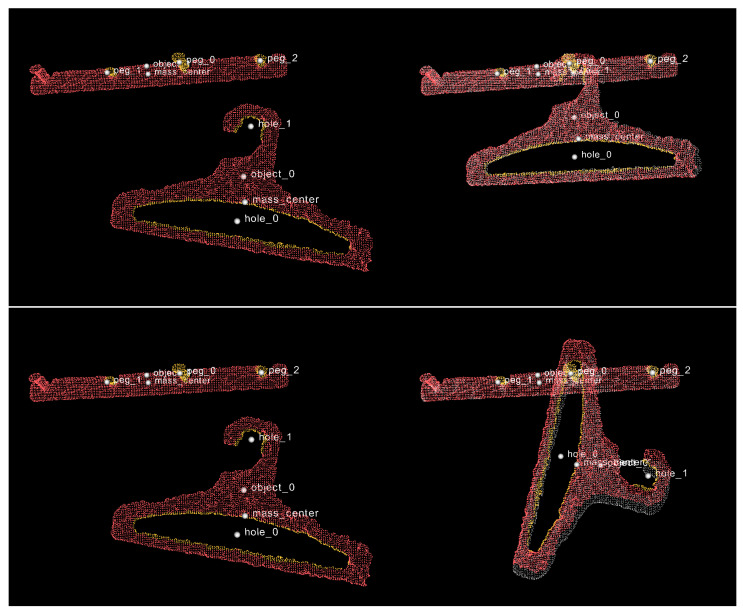
Two demonstrations, **upper** and **lower**, are input to the system. On the **left** are the point clouds representing scenes before the task execution. On the **right** are the point clouds representing scenes after the task execution. Whole objects are highlighted in red and detected object features are highlighted in yellow.

The key element of our approach is to focus on object features rather than on whole objects. Therefore, we employed a number of feature detection routines (Figure 1B) to detect various significant parts of the objects involved in the task. We developed a procedural hole detector that looks for apertures in objects. Our goal was to detect holes from a functional perspective; therefore, we did not require the aperture to be completely closed. For instance, we can see in Figure 2 that the semi-closed space in the top section of the hanger is classified as a hole. Similarly, we created detectors for pegs (parts of the object that stick out). We also have a routine for estimating the center of mass, which is often useful for conducting certain tasks, such as deciding how to hang an item with stability (the center of mass should be below the peg).

### 3.7. Describing Relations

The next step is to describe relations between previously detected objects and their features. For this, we used a number of primitive routines(Figure 1D) designed to find meaningful but abstract relations. In the current prototype, we implemented routines for the following relations: below and inside. They work by comparing object positions and their estimated bounding boxes.

### 3.8. Task Understanding

**Figure 3:**
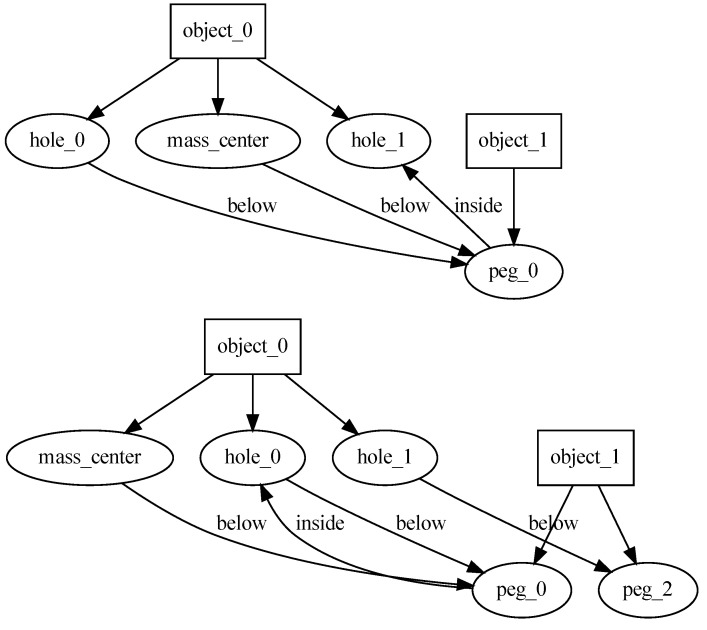
Scene descriptions in the form of a graph of feature relations. They correspond to the point clouds depicted in Figure 2.

Having all the description elements in place, the next step is to formulate hypotheses about the observed task. This is done by comparing the before-scene and after-scene graphs (Figure 3). The system looks for relations created during the task execution and turns them into constraints that must be satisfied for the task to be successful. This knowledge about task requirements can also be expressed in the form of a graph. Execution of such a task means grounding its symbols in the new scene facing the robot.

### 3.9. Skill Learning

At this stage, it is likely that we end up with more constraints than necessary. Such redundancy is not a problem if we always use the same objects. However, ideally, the system should infer the minimal set of constraints that might be useful for many different objects. We can facilitate this by providing another demonstration of the same task but with a different object. In our example scenario, we provide a second demonstration with the same hanger but hanging it differently: this time using the aperture in its center rather than the dedicated hook. (For some demonstrations, we had to manually provide the transformation of objects between before-scene and after-scene, as our object detection routine could not always cope with this automatically. Similarly, the “inside” relation detector did not work properly, so we had to enter that information manually into the scene graph.)

The result of skill learning is a generalized set of rules that should be satisfied for any hanging task to be successful. These rules can be viewed as a table or as a graph.

### 3.10. Formal Characterization

Definitions 1 outline a formalism of our graph-based knowledge representations and functions used to produce this representation. The knowledge corpus (6) consists of known entity and relation types (1, 2). Entities are represented as nodes on knowledge graphs, relations are represented as graph edges. Nodes carry information (3) about the scene parts they represent. Graphs are made of entity (4) and relation (5) instances: they constitute scene (8) and task (9) representations, which together form the knowledge base (7).

Using this formalism we can describe our task understanding algorithm (Algorithm 1). It uses several functions (Definition 1, 12–17) for intermediate steps of processing point clouds and graph operations. The result of the algorithm is a task description ready to be grounded and used.

**Definition** **1.**
(1)E−Asetofknownsceneentitytypes(2)R−Asetofknownentityrelationtypes(3)P−Entityproperties:id,position,etc.(4)Ei:={Et,Ep},Et∈E,Ep∈PEntityInstance(5)Ri:={Rt,Ei1,Ei2},Rt∈R,Ei1,Ei2∈EiRelationInstance(6)Kc:={E,R}KnowledgeCorpus(7)Kb:={Sr1,Sr2,Tr}KnowledgeBase(8)Sri:={Sei,Sri},Sei∈Ei,Sri∈Rii∈{1,2}SceneRepresentation(9)Tr:={St,Sr},St∈E,Sr∈RTaskRepresentation(10)G(Tr)⟶Srt,Srt−DesiredscenestateTaskKnowledgeGrounding(11)Pc−Apointcloud(12)PreProcess(Pc)⟶PcPre-processing(13)DetectObjects(Pc)⟶EiObjectdetection(14)DetectFeatures(Ei)⟶EiFeaturedetection(15)DetectRelations(Ei)⟶RiRelationdetection(16)DescribeScene(Ei,Ri)⟶SrScenedescription(17)DescribeTask(Sr1,Sr2)⟶TrTaskdescription

**Algorithm 1** Task understanding algorithm **Input**  $Pc_{1}$ point cloud of the before-scene  $Pc_{2}$ point cloud of the after-scene **Output**  Tr Task description
1:

$\begin{array}{ll} Pc_{1}\leftarrow PreProcess\left(Pc_{1}\right),Pc_{2}\leftarrow PP\left(Pc_{2}\right) & \end{array}$

2:

$\begin{array}{ll} Se_{1}\leftarrow \left\{\right\},Sr_{1}\leftarrow \left\{\right\},Se_{2}\leftarrow \left\{\right\},Sr_{2}\leftarrow \left\{\right\} & \;\;\;\;\;\;\;\;\;\;\;\;\;\;\;▹\;\mathrm{empty}\;\mathrm{sets} \end{array}$

3:

$\begin{array}{ll} Se_{1}+=DetectObjects\left(Pc_{1}\right),Se_{2}+=DetectObjects\left(Pc_{2}\right) & ▹\;\mathrm{adding}\;\mathrm{elements}\;\mathrm{to}\;\mathrm{sets} \end{array}$

4:

$\begin{array}{ll} Se_{1}+=DetectFeatures\left(Se\right),Se_{2}+=DetectFeatures\left(Se\right) & \end{array}$

5:

$\begin{array}{ll} Sr_{1}+=DetectRelations\left(Se\right),Sr_{2}+=DetectRelations\left(Se\right) & \end{array}$

6:

$\begin{array}{ll} Sd_{1}\leftarrow DescribeScene\left(Se_{1},Sr_{1}\right),Sd_{2}\leftarrow DescribeScene\left(Se_{2},Sr_{1}2\right) & \end{array}$

7:

$\begin{array}{ll} Tr\leftarrow DescribeTask(Sd_{1},Sd_{2}) & \end{array}$




### 3.11. Computation Complexity

The reference hardware used to report computation times uses a 2.5 GHz Quad-Core Intel Core i7 with 16 GB 1600 MHz DDR3 memory and an SSD hard drive.

Most of the computation complexity comes from opening and processing raw point clouds before they can be used in reasoning mechanisms. Pre-processing was done once for every point cloud. In our main experiment, we used a total of four point clouds to generate a good quality task description. Pre-processing involves statistical outlier removal, downsampling, computing normal vectors for each point, fitting, and removing the plane using RANSAC fitting. The average (collected over 10 runs) pre-processing time for one point cloud is 4465 ms. Each raw point cloud consists of about 786,000 points.

All feature recognition modules that use PCL library functions, which currently all of them do, are subject to fine-tuning, which may dramatically change the speed of execution depending on the chosen settings. In Table 2, we list the execution times for our recognition modules.

Each relation recognition routine makes at least $n^{2}$ comparison operations, where *n* is the number of vertices in the graph.

The complexity of the task description algorithm depends on the sizes of the graphs that are built for a given pair of point clouds. The number of operations used in this algorithm can be estimated as follows:

$$N_{op}\approx NV_{b}+NV_{a}+NV_{b}\cdot NE_{b}+NV_{a}\cdot NE_{a}+2\cdot \left(NV_{b}+NV_{a}\right)$$

where:

$N_{op}$ is the number of operations.
$NV_{b}$ is the number of vertices in the before-graph.
$NV_{a}$ is the number of vertices in the after-graph.
$NE_{b}$ is the number of edges in the before-graph.
$NE_{a}$ is the number of edges in the after-graph.

The complexity of the task understanding refinement algorithm, which merges task descriptions, is similar; however, this algorithm operates on much smaller graphs:

$$N_{op}\approx NV_{t1}+NV_{t1}+NV_{t1}\cdot NE_{t1}+NV_{t2}\cdot NE_{t2}+2\cdot \left(NV_{t1}+NV_{t2}\right)$$

where:

$N_{op}$ is the number of operations.
$NV_{t1}$ is the number of vertices in the first task graph.
$NV_{t2}$ is the number of vertices in the second task graph.
$NE_{t1}$ is the number of edges in the first task graph.
$NE_{t2}$ is the number of edges in the second task graph.

## 4. Experiments and Results

### 4.1. Hanging Task

**Figure 4:**
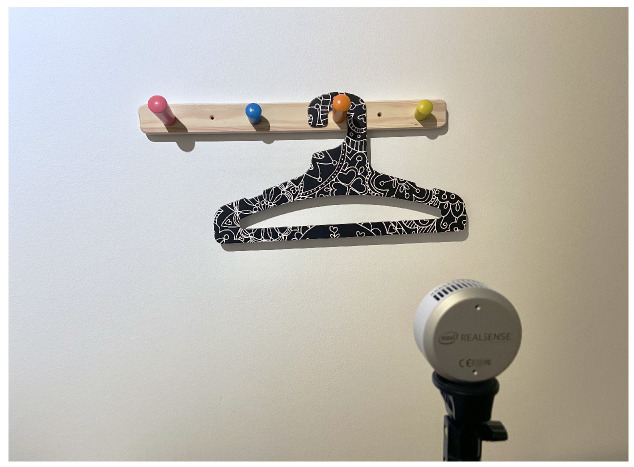
The setup. A LiDAR-based depth camera on a tripod standing in front of the experiment scene.

The following setup was used for our training experiments in the task of hanging items on pegs: A peg rail was mounted on a wall, with a depth camera (Intel RealSense L515) standing in front of it (Figure 4). In the first step, we introduced an object, a cloth hanger in this example, by holding it in front of the camera. This image was captured by the camera and stored as before-point-cloud. Next, we hung the cloth hanger on one of the pegs and took another snapshot as after-point-cloud. The process was repeated but with the following change: the hanger was hung not by its hook but by its central aperture. All the point clouds captured in these steps were then given as input to the task-understanding algorithm.

**Figure 5:**
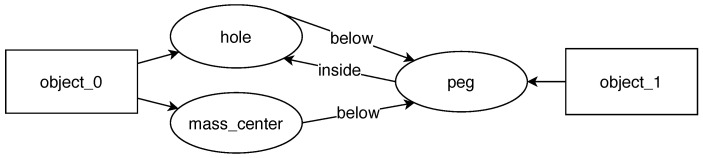
A graph representing learned skill requirements.

The results of the learning process are presented in two different ways: as a table (Table 3) and as a graph (Figure 5). It can be noted that the last rule in the table is somewhat questionable. It is there because both our demonstrations include that relation. This suggests that there is potential for creating a more universal set of rules by providing more diverse demonstrations.

### 4.2. Stacking Task

**Figure 6:**
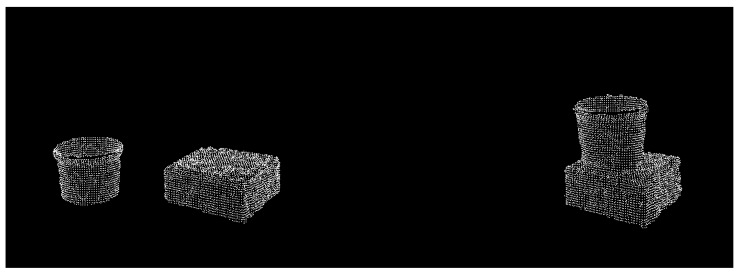
Point clouds (pre-processed) of the item stacking task. **Left**: before the demonstration, **right**: after the demonstration.

To further demonstrate the flexibility of our proposed knowledge representation and scene understanding system, we performed another experiment with the task of stacking one item on top of another. For example, we have a mug and a box laying on the table (see Figure 6). During the demonstration, we picked up the mug and placed it on top of the box. We performed this demonstration two times.

For this task, the core of the reasoning system, as well as the overall processing pipeline, stayed the same as before. To produce good quality task descriptions, we added two new feature recognition routines for detecting surfaces, and object bases. Additionally, a new relation of “touching” was introduced. Irrelevant recognition modules for the “inside” relation, as well as the “hole” and “pegs” features, were turned off. (We expect that in future applications of the system, appropriate modules will be chosen depending on the characteristics of the task or a robot will autonomously test different hypotheses to prune unnecessary features from task understanding graphs.)

**Figure 7:**
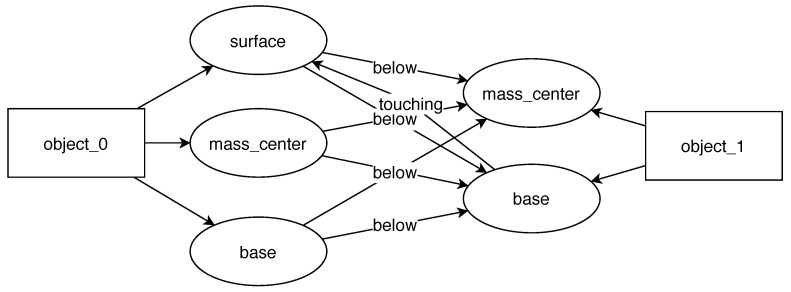
A graph representing learned skill requirements for the task of stacking items.

The result of learning the stacking task is shown in Figure 7. The system has correctly inferred that to complete this task, it must position the mug (object_1) in such a way that its base touches the surface of the box (object_0). Moreover, the center of mass of the mug must be above the surface. Two rules could be considered equivalent or redundant: these could be interchanged or used together to position the mug. Nonetheless, all three rules are correct.

### 4.3. Ablation Studies

The quality of the task description depends on the types of recognition routines available in the system. We can demonstrate that dependency by turning off different recognizers and examining the system’s output. The effects of turning off some recognition routines in the hanging task are presented in Table 4.

## 5. Discussion

**Figure 8:**
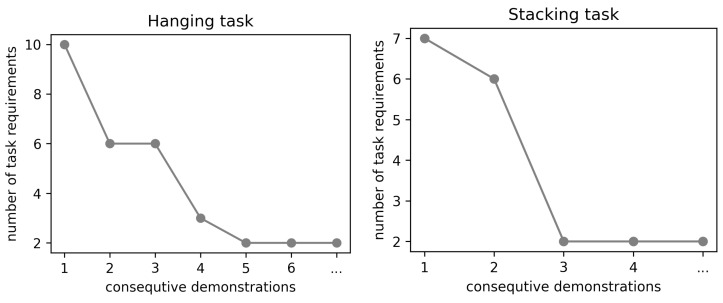
Plots of the learning process in the task of hanging (**left**), and stacking (**right**). The number of inferred task requirements decreases with each demonstration to then converge to a minimum.

To test the learning efficiency of our system, we ran tests where the system had all recognition modules turned on, some of which were irrelevant to the task at hand. By providing multiple demonstrations, we were able to prune all unnecessary task rules and keep only the relevant ones. As seen in Figure 8, the hanging task converged to two essential rules after five demonstrations. The stacking task converged just after two demonstrations. In general, the more variations there are in different demonstrations, the faster the system learns.

There is a requirement that must be met for the learning process to be successful: all features and relations crucial for the task must be successfully recognized in each demonstration. This is a limitation that could be addressed in several ways. For instance, if an entity or relation is present in most of the demonstrations, we could assume that it should be there, even though some demonstrations lack them.

## 6. Conclusions and Future Research

The knowledge representation used in this paper, while drawing inspiration from more comprehensive knowledge representation systems can be thought of as a particular instantiation of a graph-based knowledge representation scheme that is especially well-suited for skill learning and adaptation in robots. Contrary to many contemporary graph-like representations, it focuses not on objects themselves but their constituent parts, features, and their affordances—‘objects’ are merely bags for grouping these affordances under one label.

The computer vision system presented here demonstrates how this knowledge representation can be used for generalizing skills and explaining them to a human. This highly modular system can form descriptions of scenes perceived through a depth camera, and infer requirements that must be satisfied for completing a task successfully.

The scene understanding approach used by our system can be integrated into a bigger robotic system as it works with real objects captured by a depth camera from one angle.

Future extensions of our proposed system include both improvements to the current system as well as developing companion systems for task planning and execution on a robotic platform.

Our vision system could benefit from more advanced recognition techniques that include color information and better point cloud filtering or smoothing. Each recognition module of the system can be improved individually to use the state-of-the-art algorithms appropriate for it. All of the recognition modules could also benefit from adding more cameras and using a continuous point cloud registration. Our current system consists of the simplest recognition routines that worked for our dataset, as the main focus of the research presented here was on understanding the algorithms rather than computer vision techniques.

In future research, we also plan to develop a manipulation planner to execute motions on a robot, as well as develop more recognition modules to extend vision capabilities. This system could be accompanied by task planning and execution systems in order to use on a robot. In this way, a robot could perform experiments to validate hypotheses on its own, reducing the need to provide multiple demonstrations by hand. 

## Figures and Tables

**Table 1:** A comparison of some aspects of related works that can be compared with our system. Takeuchi et al. is a deep learning-based approach while Mitrevski et al. and Kazhoyan and Beetz are based on symbolic knowledge, they require some programming in order to be used in different tasks. The Kazhoyan and Beetz system offers a programmable interface instead of learning.

	Gajewski-Indurkhya	Takeuchi et al. [15]	Mitrevski et al. [3]	Kazhoyan-Beetz [11]
Works without prior knowledge of 3d models	Yes	Yes	No	No
Can generate human-understandable explanations	Yes	No	Partially	Partially
Learns from few (<10) demonstrations	Yes, ≥2	No, ≥1000	No, 25	No
Can learn different tasks	Yes	No	Manually	Manually

**Table 2:** Average feature recognition routine run times across five trials.

Object Detection	Pegs Detection	Holes Detection	Object Properties Detection
165 ms	11 ms	73 ms	43 ms

**Table 3:** A set of rules representing learned skill requirements.

Object 1	Feature Type	Required Relation	Object 2	Feature Type
object_1	peg	inside	object_0	hole
object_0	mass_center	below	object_1	peg
object_0	hole	below	object_1	peg

**Table 4:** The effects of turning off certain recognition routines.

Recognized Feature or Relation	Effect on Task Description
Below relation	There is no information on how to position the cloth hanger center of mass relative to the peg, which may result in an unstable hang if the task is to be executed.
Inside relation	The requirement of placing the cloth hanger aperture in such a way that the peg is inserted inside of it is missing from the task description.
Hole entity	The resulting task description only requires positioning the hanger’s center of mass somewhere below the peg
Peg entity	The resulting task description is completely empty. The system found no way to describe the task without the notion of a peg.

## Data Availability

Point clouds used in this work are available online at: https://github.com/lubiluk/halepensis/tree/main/data/ (accessed on 12 August 2022).
